# Latest Advancements in Using Fe_3_O_4_@graphene Oxide Nanocatalyst for the Hydrogenation of Nitroarenes

**DOI:** 10.1002/open.70157

**Published:** 2026-02-28

**Authors:** Sara Payamifar, Majid Abdouss, Hamideh Sarreshtehdar Aslaheh, Ahmad Poursattar Marjani

**Affiliations:** ^1^ Department of Chemistry Amirkabir University of Technology Tehran Iran; ^2^ Department of Organic Chemistry Faculty of Chemistry Urmia University Urmia Iran

**Keywords:** amines, Fe_3O__4_ nanoparticles, graphene oxide, nanocatalyst, nitroarenes, reduction

## Abstract

Reducing nitroarenes to aromatic amines is a valuable method in both industrial and synthetic organic chemistry for producing amine compounds. Recently, magnetite‐based nanocatalysts have attracted attention as promising, eco‐friendly alternatives to conventional noble‐metal catalysts in this transformation. Magnetite nanoparticles, with their large surface area, strong catalytic performance, and inherent magnetic properties, enable easy separation and reuse, significantly supporting the sustainability of the process. Fe_3_O_4_@graphene oxide nanocatalysts are regarded as highly promising due to their straightforward synthesis, affordability, excellent superparamagnetic behavior, good biocompatibility, and low toxicity. Utilizing environmentally friendly reducing agents such as sodium borohydride or H_2_, alongside these adaptable nanocatalysts, enables fast and selective transformation of nitroarenes under moderate reaction conditions. This study emphasizes the promise of Fe_3_O_4_@graphene oxide magnetic nanocatalysts as an economical, recyclable, and environmentally friendly catalyst for the hydrogenation of nitroarenes, offering a valuable approach toward a more sustainable chemical industry. This review provides an overview of the synthesis and applications of Fe_3_O_4_@graphene oxide magnetic nanocatalysts, highlighting their effectiveness and eco‐friendliness in the hydrogenation of nitro compounds, with coverage extending through 2025.

## Graphical abstract

This review provides a comprehensive summary of recent advances in Fe_3_O_4_@graphene oxide magnetic nanocatalysts for the reduction of nitroarenes. The reduction of nitroarenes yields valuable intermediates that serve as key building blocks in the synthesis of agrochemicals and pharmaceuticals. Fe_3_O_4_@graphene oxide magnetic nanocatalysts are expected to enable the industrial removal of hazardous nitro compounds in the future.

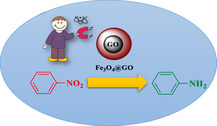

**Abbreviations**
APTES: 3‐Aminopropyltriethoxysilane
4‐AP: 4‐Aminophenol
4‐NP: 4‐Nitrophenol
ABC: Ammonium bismuth citrate
BET: Brunauer–Emmett–Teller
CNTs: Carbon nanotubes
Chit: Chitosan
EDX: Energy‐dispersive X‐ray
EPA: Environmental protection agency
FT‐IR: Fourier transform infrared
GLIP: Gas‐liquid interfacial plasma
GO: Graphene oxide
ICP: Inductively coupled plasma
ICP‐AES: Inductively coupled plasma‐atomic emission spectroscopy
ICP‐OES: Inductively coupled plasma optical emission spectroscopy
IL: Ionic liquid
MNPs: Magnetite nanoparticles
PEI: Polyethyleneimine
PXRD: Powder X‐ray diffraction
RGO: Reduced graphene oxide
SEM: Scanning electron microscopy
NaBH_4_
: Sodium borohydride
TGA: Thermogravimetric analysis
TEM: Transmission electron microscopy
TOF: Turnover frequency
TON: Turnover number
Uv‐Vis: Ultraviolet/visible spectroscopy
VSM: Vibrating‐sample magnetometer
XRD: X‐ray diffraction
XPS: X‐ray photoelectron spectroscopy

## 1 Introduction

Nitrobenzenes and their derivatives serve as key organic starting materials in chemical synthesis. They are vastly used in the manufacture of dyes, pigments, plastics, pharmaceuticals, pesticides, fungicides, explosives, and industrial solvents [1, 2, 3, 4, 5]. Nevertheless, these substances pose serious risks to both human health and the environment [6, 7]. Among nitroaromatic derivatives, 4‐nitrophenol (4‐NP) is particularly persistent and difficult to degrade, and it is commonly found in industrial wastewater from chemical manufacturing and agricultural effluents [8]. This compound is highly toxic and, because of its low biodegradability, strong stability, and high water solubility, it remains persistent in both environmental and biological systems. Even at low concentrations, it can cause severe harm to the central nervous system, kidneys, and liver of humans and creatures [9, 10, 11, 12]. Because of these concerns, the United States Environmental Protection Agency (EPA) has designated 4‐NP as a “priority pollutant.” As a result, it is crucial to monitor its environmental behavior and develop effective methods for its removal from wastewater prior to discharge. Numerous approaches have been developed for eliminating 4‐NP from polluted water, including photocatalytic degradation [13], microwave‐assisted catalytic oxidation [14], catalytic reduction [15], electrochemical processes [16], the electro‐Fenton technique [17], and biodegradation [18], among others. Of the methods listed above, catalytic reduction of 4‐NP to 4‐aminophenol (4‐AP) in water is a helpful, economical, and green approach for this application [19]. 4‐AP is a vital intermediate in the production of numerous analgesic medications. In addition, it is widely utilized as a corrosion inhibitor, photographic developer, hair dye agent, and anticorrosion lubricant [20, 21]. Therefore, developing an efficient and eco‐friendly catalytic system for converting 4‐NP to 4‐AP is highly significant.

Lately, magnetic nanoparticles (MNPs) have attracted increasing interest owing to their broad range of applications, including environmental remediation, magnetic resonance imaging (MRI), drug delivery, magnetic inks, energy storage, catalysis, and magnetic fluids [22, 23, 24, 25, 26, 27, 28, 29, 30]. In organic chemistry, employing MNPs as catalysts or supports for catalytic systems in organic reactions has become a rapidly expanding area of research. This interest stems from the fact that MNPs can be conveniently separated and reused using an external magnet, making them attractive from both environmental (green chemistry) perspectives and economic [31, 32, 33, 34, 35, 36].

Among the different kinds of MNPs, Fe_3_O_4_ NPs have received greater focus owing to their distinctive characteristics, including simple synthesis, cost‐effectiveness, nonpoisonous nature, biological compatibility, strong superparamagnetic behavior, and highly active surfaces that facilitate the adsorption and immobilization of ligands, metals, and catalytic components [37, 38, 39, 40, 41]. However, because of their elevated surface energy, Fe_3_O_4_ NPs tend to aggregate and form larger bulk structures quickly. To address this issue and enhance their chemical stability while also gaining benefits such as versatile surface modification, these nanoparticles are commonly coated with a variety of organic or inorganic substances, with graphene oxide (GO) being a recent choice [42, 43, 44, 45, 46].

**SCHEME 1:**
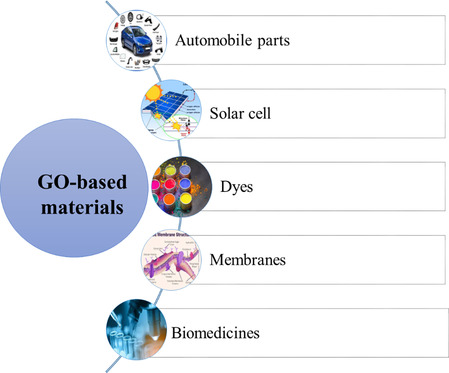
The different applications of GO‐based materials.

GO, a fascinating carbon‐based nanomaterial, is an excellent environmentally friendly option for stabilizing Fe_3_O_4_ MNPs due to its remarkable features, including mechanical strength, a high surface area, and adjustable electrical and optical properties [47, 48, 49, 50]. Various applications of GO‐based materials are exhibited in Scheme 1. The high abundance of various oxygen‐containing functional groups on GO's large surface area, such as epoxy, hydroxyl, and carboxylic acid units, not only ensures outstanding dispersibility in water but also provides effective sites for anchoring desired substances and enabling additional chemical modifications [51, 52, 53].

Fe_3_O_4_@graphene oxide magnetic nanocatalysts represent a novel and efficient class of nanocomposites that combine the unique features of Fe_3_O_4_ NPs with those of GO. Fe_3_O_4_ NPs are valued for their simple synthesis, low cost, biocompatibility, and excellent superparamagnetic properties, which enable easy magnetic separation and recovery of the catalyst, a crucial factor for economic and environmental sustainability. However, their high surface energy often leads to aggregation, thereby reducing their activity. Coating Fe_3_O_4_ nanoparticles with graphene oxide overcomes this drawback by providing chemical stability, preventing aggregation, and offering a large surface area rich in oxygen‐containing functional groups (such as epoxy, hydroxyl, and carboxylic acids). These groups not only improve aqueous dispersibility but also serve as effective anchoring sites for catalytic species or further modifications. The synergy between Fe_3_O_4_ and graphene oxide enhances catalytic performance, making Fe_3_O_4_@GO an excellent magnetic nanocatalyst, especially for organic reactions such as the reduction of nitroarenes. The magnetic nature allows easy catalyst recovery using external magnets, reducing waste and cost. Additionally, graphene oxide improves electron transfer and catalytic efficiency. This composite has demonstrated excellent catalytic activity, high stability across multiple reaction cycles, and environmental friendliness, positioning it as a promising system for the catalytic reduction of nitroarenes to valuable amines, such as 4‐aminophenol, which are essential intermediates in pharmaceuticals and other industrial applications [54, 55, 56, 57].

**FIGURE 1:**
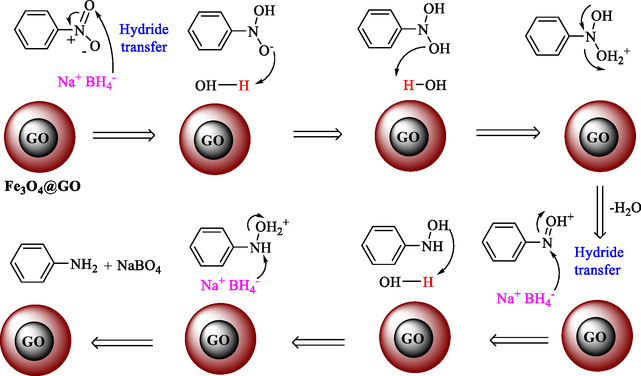
The mechanistic steps for the reduction of nitroarenes by Fe_3_O_4_@GO.

The reduction of the nitroaromatic compound occurs via hydride transfer from NaBH_4_ to the nitro group, producing a nitroso group, which then reacts with an additional hydride to form the amine product (Figure 1) [8, 9].

The mechanism comprises the subsequent stages:

1. Hydride transfer: the reduction process begins with the transfer of hydride ions from NaBH_4_ to the nitro group of the aromatic compound, transforming it to a nitroso intermediate.
2. Construction of hydroxylamine: subsequent hydride transfers further reduce the nitroso group to construct a hydroxylamine intermediate.
3. Final reduction to amine: additional hydride ions reduce the hydroxylamine to the final amine product.

There has been increasing interest in modifying MNPs with various ligands and materials to improve the efficiency of the catalysts made from them. A key advantage of such magnetic nanoparticles is their ease of recovery and separation via magnetic fields. Magnetite‐based nanocatalysts offer an environmentally friendly and economical approach to the reduction of nitroarenes, combining strong magnetic properties with versatile functionalization, which is especially valuable for sustainable chemical applications across numerous industries. This review highlights the latest advancements in utilizing Fe_3_O_4_@graphene oxide nanocatalysts for the reduction of nitroarenes.

## 2 The Usage of Fe_3_O_4_@GO Nanocatalysts for Reducing the Nitroaromatic Derivatives

In summary, Fe_3_O_4_@GO nanocatalysts combine the magnetic retrievability and biocompatibility of Fe_3_O_4_ with the chemical stability and functional versatility of graphene oxide, creating an efficient, recyclable, and environmentally benign catalytic system for reduction reactions of nitroarenes.

A straightforward, effective, and broadly applicable method was reported by Li et al. for selectively anchoring noble metal nanoparticles (Pt, Pd, or bimetallic PtPd, 3–5 nm) onto magnetite/graphene composites [58]. The amino acid L‐lysine, containing both amine (–NH_2_) and carboxyl (–CO_2_H) groups, served as a vital linking agent between the noble metals and the Fe_3_O_4_/graphene support. The resulting composites were thoroughly characterized using TEM, XRD, EDX, and XPS, revealing that the noble metals were predominantly distributed on the magnetite surfaces. These materials demonstrated excellent stability and could be easily separated magnetically, making them well‐suited for reuse in liquid‐phase catalytic reactions. Notably, the PtPd‐based composites showed superior catalytic activity and strong resistance to deactivation during the NaBH_4_‐mediated hydrogenation of 4‐NP to 4‐AP. The efficient synthesis route offered promising potential for industrial applications, particularly where catalyst recovery and reuse are essential to minimize costs and environmental impact.

In 2013, He et al. reported the preparation, characterization, and catalytic application of a Fe_3_O_4_@GO nanocomposite as a magnetically recyclable, highly efficient catalyst for the hydrogenation of nitroarenes to aromatic amines using hydrazine hydrate [59]. The nanocomposite was prepared via a straightforward co‐precipitation procedure, using FeCl_3_ · 6H_2_O and FeSO_4_ · 7H_2_O with GO in ethanol under basic conditions. Different Fe_3_O_4_:GO mass ratios (2–20) were tested; the 10:1 ratio showed optimal performance. Uniform dispersion of ∼12 nm Fe_3_O_4_ nanoparticles on GO sheets and high surface area (137 vs. 58 m^2^/g for bare Fe_3_O_4_NPs) confirmed by XRD, FTIR, TEM, and BET analyses. GO prevented nanoparticle aggregation and enhanced catalytic properties. The Fe_3_O_4_@GO catalyst achieved 99.2% yield of aniline from nitrobenzene in 18 min, with a TOF of 3.63 min^−1^, 45 times higher than commercial Fe_3_O_4_ NPs. The composite worked effectively at a low catalyst load (3.1 wt%) and with lower hydrazine usage than prior methods. Catalyst dosage, hydrazine amount, solvent, and temperature were systematically studied. Ethanol at reflux with 3.6 equiv. Hydrazine was found to be optimal. Other solvents, like toluene and isopropanol, also showed promising results. The catalyst was easily recovered with a magnet and maintained high activity after six cycles, demonstrating excellent stability. The catalyst was effective for a range of nitroarene substrates (Table 1), including substituted nitrobenzenes, demonstrating broad applicability and superior performance compared with traditional catalysts. The Fe_3_O_4_@GO composite is a cost‐effective, productive, and reusable nanocatalyst for the hydrogenation of nitroarenes. The incorporation of graphene oxide significantly enhances catalytic performance by preventing nanoparticle aggregation and improving substrate interaction.

**TABLE 1:** Reduction of some nitroarenes using Fe_3_O_4_@GO.

Entry	Nitroarenes	Product	Time, min	Yield, %
1	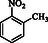	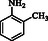	40	98.8
2	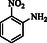	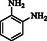	45	97.5
3	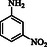	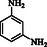	80	97.1
4	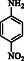	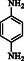	95	82.5
5	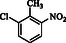	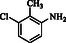	20	99.2

**SCHEME 2:**
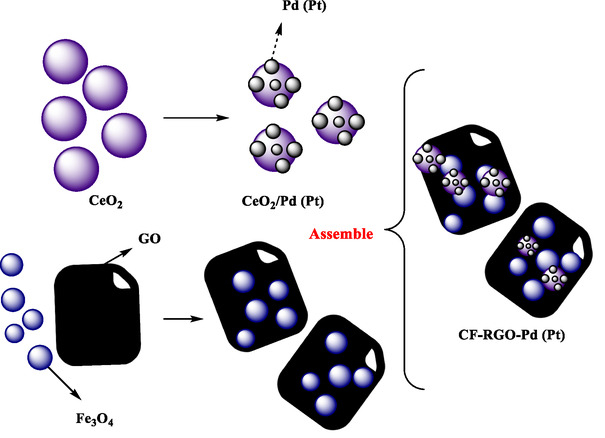
Schematic synthesis process of CF‐RGO‐Pd (Pt) hybrids.

Wang et al. reported a novel and efficient strategy for fabricating highly active and magnetically recyclable Pd and Pt nanoparticle‐based catalysts [60]. The authors employed a hierarchical self‐assembly process to create hybrid nanocomposites composed of noble metals (Pd or Pt) as active catalytic sites, CeO_2_ as a noble‐metal support, Fe_3_O_4_ nanoparticles for magnetic separation, reduced graphene oxide (RGO) as a structural linker, and conductive support. TEM, XPS, FTIR, XRD, and VSM analyses confirmed strong interactions, uniform dispersion, and magnetic properties for easy recovery. These catalysts, named CF‐RGO‐Pd and CF‐RGO‐Pt, demonstrated excellent catalytic performance in the selective reduction of nitrophenol derivatives (*p*‐, *m*‐, and *o*‐ nitrophenol), with TOFs up to 8500 h^−1^. The combination of CeO_2_ with noble‐metal nanoparticles on graphene‐coated magnetic substrates provides synergistic effects that enhance catalytic efficiency by improving electron transfer, enhancing reactant adsorption, and preventing nanoparticle aggregation (Scheme 2). The magnetic Fe_3_O_4_ enabled simple recovery of the nanocatalyst using an external magnet, enabling reusability up to 10 cycles with negligible activity loss. RGO facilitated uniform dispersion and intense interactions with substrates, thereby improving contact with active catalytic centers. The self‐assembly process used here is more straightforward and versatile than conventional layer‐by‐layer or Janus particle approaches. This research demonstrated a general, scalable, and effective approach to designing multifunctional catalysts with high activity, stability, and recyclability.

These hybrid nanocatalysts offered a promising platform for green, sustainable chemical processes, particularly in environmental and biological applications. A comparison of the catalytic activity of these magnetic noble‐metal nanocatalysts with other reports is displayed in Table 2.

**TABLE 2:** A comparison of the catalytic activity of these magnetic noble‐metal catalysts with other reports.

Catalyst	Nitroarenes	Yield, %	**TOF, h** ^**−1**^	Ref.
Pt/Fe_3_O_4_/RGO	4‐NP	100	5294	[58]
Pd/Fe_3_O_4_/RGO	4‐NP	100	2500	[58]
PtPd/Fe_3_O_4_/RGO	4‐NP	100	4615	[58]
Fe_3_O_4_@SiO_2_@Au@m‐SiO_2_	4‐NP	100	1396	[61]
CF‐RGO‐Pt	2‐NP	100	1000	Currently discussed report
CF‐RGO‐Pd	4‐NP	100	8500	Currently discussed report
CF‐RGO‐Pd	2‐NP	100	6800	Currently discussed report
CF‐RGO‐Pt	4‐NP	100	1320	Currently discussed report
CF‐RGO‐Pt	3‐NP	100	730	Currently discussed report
CF‐RGO‐Pd	3‐NP	100	5500	Currently discussed report

Feng et al. developed and evaluated a novel graphene‐Fe_3_O_4_ (G‐Fe_3_O_4_) magnetic nanocomposite as a helpful, recyclable catalyst for the hydrogenation of nitroarenes to anilines using hydrazine hydrate as the hydrogen source [62]. G‐Fe_3_O_4_ was prepared via a chemical co‐precipitation procedure, yielding Fe_3_O_4_ nanoparticles (∼25–50 nm) uniformly distributed on graphene sheets. The nanocomposite demonstrated high catalytic activity in converting a wide range of nitroaromatic compounds into their corresponding amines. The catalyst efficiently hydrogenated various nitroarenes to corresponding anilines with high selectivity and minimal byproducts. The reactions proceed smoothly under mild conditions, such as room temperature or moderate heating, often without the need for noble metals, using a nanocatalyst 5%. The yields ranged from 82% to 92%, with excellent chemoselectivity, even in the presence of functional groups such as halogens and carboxylic acids (Table 3). The catalyst was easily separated using a magnet and recycled for five runs with no substantial reduction of performance or Fe leaching. The graphene support prevented nanoparticle aggregation, enhanced substrate adsorption, and promoted electron transfer, boosting catalytic performance.

**TABLE 3:** Hydrogenation of nitroarene derivatives using G‐Fe_3_O_4_.

Entry	Nitroarenes	Product	T,°C	Time, min	Yield, %
1	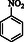	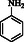	70	20	88.4
2	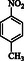	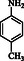	70	85	89
3	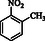	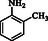	70	360	83.9
4	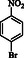	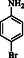	70	80	90.5
5	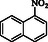	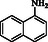	70	255	91.9
6	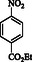	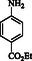	Reflux	240	91.4
7	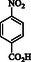	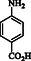	Reflux	300	90.5
8	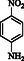	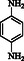	Reflux	300	82.4

*Note:* Reaction condition: Molar ratio of nitroarenes to hydrazine hydrate (1:2) and catalyst (5%).

Additionally, reducing GO to partially RGO during synthesis improves catalyst stability and activity. The G‐Fe_3_O_4_ magnetic nanocomposite was an extremely efficacious, reusable, and eco‐friendly nanocatalyst for the reduction of nitroarenes. Its magnetic recoverability, high selectivity, and cost‐effectiveness made it promising for industrial and green chemistry applications.

In 2014, Gupta et al*.* reported the preparation, characterization, and uses of a recyclable magnetic catalyst, Fe@Au‐ATPGO, composed of Fe@Au core‐shell NPs anchored on GO, for the catalytic hydrogenation of nitrophenol compounds (specifically 4‐NP and 2‐NP) using NaBH_4_ [63]. This material was analyzed by XPS and TEM, confirming the spherical nature and uniform distribution of the nanoparticles, with average diameters of 10–12 nm. XPS analysis confirmed successful functionalization and chemical attachment of the nanoparticles to GO. The nanocatalyst was applied to reduce 4‐NP and 2‐NP in the existence of NaBH_4_. Kinetic and thermodynamic studies showed that 4‐NP was reduced faster than 2‐NP. Activation energies for the reduction were determined to be 2.33 kcal/mol for 4‐NP and 3.16 kcal/mol for 2‐NP. The catalyst, being magnetic, could be easily separated from the reaction mixture using a magnet and recycled multiple times with no significant decrease in performance. This property enhances its practical and economic benefits for industrial applications. The nanocatalyst was magnetically recoverable within 15 s. It was successfully recovered for 10 runs with negligible decline in activity, demonstrating excellent stability and economic viability.

In 2015, Wang et al. reported a simple method for synthesizing magnetic hybrid nanocatalysts comprising bismuth (Bi) nanoparticles and Fe_3_O_4_ NPs supported on reduced graphene oxide (RGO) [64]. The main purpose was to develop an efficient, reusable, and easily recoverable catalyst for the hydrogenation of 4‐NP, a significant environmental contaminant, to 4‐AP. Fe_3_O_4_ nanoparticles were synthesized by co‐precipitating Fe^2+^ and Fe^3+^ ions, while Bi NPs were produced through redox reactions between NaBH_4_ and ammonium bismuth citrate (ABC), all in the existence of GO and soluble starch as dispersants. The resulting Bi‐Fe_3_O_4_@RGO hybrids were purified and confirmed using various techniques. TEM image revealed Fe_3_O_4_ NPs (∼5 nm) and Bi NPs (10–20 nm) well‐dispersed on the RGO sheets. XRD and FT‐IR verified the successful construction of the hybrids and the reduction of GO. EDX data verified the composition and distribution of Bi, C, Fe, and O. The Bi‐Fe_3_O_4_@RGO hybrids exhibited high catalytic performance for the hydrogenation of 4‐NP to 4‐AP in the presence of NaBH_4_, with a first‐order rate constant of 0.00808 s^−1^. Due to their magnetic properties, the catalysts can be readily isolated from the reaction mixture using an external magnet. This nanocatalyst retained its activity for at least five recycling cycles, demonstrating excellent reusability. This work highlighted the potential of magnetic, graphene‐supported bismuth‐iron oxide hybrids for green chemistry applications, specifically for efficient, recoverable catalysis in water treatment and environmental protection.

**SCHEME 3:**
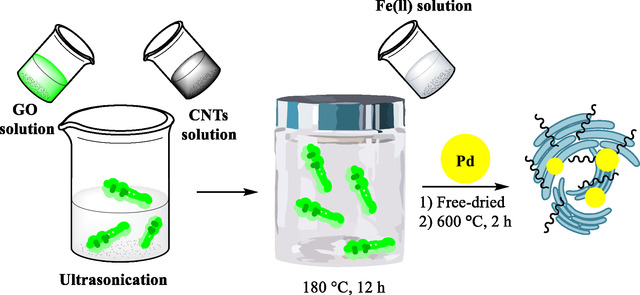
The synthesis process for GO/CNT–Fe_3_O_4_‐supported Pd nanoparticles.

In 2016, Yang et al. explained the development and characterization of a new magnetically separable nanocatalyst composed of Pd NPs supported on a hybrid of GO, carbon nanotubes (CNTs), and Fe_3_O_4_ NPs [65]. The focus was on achieving efficient, reusable catalysis for the hydrogenation of nitroarenes (such as nitrobenzene) and on catalytic C–H activation reactions, both performed in water under mild conditions. GO and CNTs are combined to create a composite that leverages the high surface area and stability of both materials but minimizes their tendency to aggregate or restack. Fe_3_O_4_ magnetic NPs are incorporated via a hydrothermal method. Their magnetic nature enables easy recovery of the catalyst with a magnet and helps prevent material aggregation. Pd nanoparticles are then uniformly deposited onto the GO/CNT‐Fe_3_O_4_ support utilizing a gas‐liquid interfacial plasma (GLIP) procedure. Several ratios of GO to CNT are explored to optimize the catalyst structure and performance (Scheme 3).

Techniques including TEM, SEM, XRD, and XPS confirm the nanocomposite structure and nanoparticle dispersion. Pd nanoparticles were found to be monodisperse with sizes around 4 nm. The composite exhibited well‐defined crystalline phases and desired chemical compositions.

The catalyst showed excellent performance in hydrogenating nitroarenes to anilines (important intermediates for pharmaceuticals and dyes) in water at 60°C under 1 atm of hydrogen gas, in green, mild conditions (Table 4). The catalysts were also active for C–H functionalization reactions. The magnetic property enabled easy and efficient catalyst isolation from the reaction mixture using a simple magnet. The catalysts were recovered after several cycles with minimal drop in performance, confirming excellent stability and reusability. Combining GO, CNTs, and Fe_3_O_4_ overcame common catalyst support issues such as aggregation and restacking, while providing high surface area and stability. This catalyst enables reactions in water and uses hydrogen gas (an environmentally benign reducing agent) under mild conditions (atmospheric pressure, moderate temperature), promoting chemical processes aligned with green chemistry principles. The ease of magnetic separation addresses a common drawback in nanocatalyst recovery.

**TABLE 4:** The hydrogenation of nitroarenes using the Pd‐3 nanocatalyst.

Entry	**Ar‐NO**_**2**_	**Ar‐NH**_**2**_	Time, h	Yield, %
1	8‐Nitroquinoline	8‐Aminoquinoline	3	99
2	3‐O_2_NC_6_H_4_NO_2_	3‐H_2_NC_6_H_4_NH_2_	12	99
3	4‐O_2_NC_6_H_4_COCH_3_	4‐H_2_NC_6_H_4_COCH_3_	12	94
4	4‐O_2_NC_6_H_4_CN	4‐H_2_NC_6_H_4_CN	12	90
5	2‐O_2_NC_6_H_4_CO_2_H	2‐H_2_NC_6_H_4_CO_2_H	12	90
6	4‐O_2_NC_6_H_4_CO_2_H	4‐H_2_NC_6_H_4_CO_2_H	12	92
7	2‐O_2_NC_6_H_4_OH	2‐H_2_NC_6_H_4_OH	24	87
8	4‐O_2_NC_6_H_4_OH	4‐H_2_NC_6_H_4_OH	24	91
9	4‐O_2_NC_6_H_4_CO_2_CH_3_	4‐H_2_NC_6_H_4_CO_2_CH_3_	12	93
10	4‐MeOC_6_H_4_NO_2_	4‐MeOC_6_H_4_NH_2_	3	99
11	4‐MeC_6_H_4_NO_2_	4‐MeC_6_H_4_NH_2_	3	88

*Note:* Reaction conditions: Pd‐3 (1 mol%), nitroaromatic compounds (0.5 mmol), H_2_ (1 atm), and H_2_O (2 mL).

Dabiri et al. reported the synthesis, characterization, and catalytic applications of a novel nanocomposite, Fe_3_O_4_@RGO@Au@C, featuring a magnetic Fe_3_O_4_ core, RGO as an inner shell, a layer of gold nanoparticles (Au NPs), and an outer carbon (C) shell [66]. The double‐shelled design (RGO inner shell, carbon outer shell) served as a protective capsule, stabilizing Au NPs and preventing aggregation and leaching. The Fe_3_O_4_ core enables easy catalyst recovery using a magnet. The composite was synthesized via a multistep process involving solvothermal and hydrothermal methods, surface functionalization, and carbonization. Techniques such as TEM, SEM, XRD, XPS, ICP‐OES, TGA/DTA, and magnetic property measurements confirmed the catalyst's structure, morphology, composition, and magnetic behavior. The catalyst efficiently catalyzed the hydrogenation of various nitroaromatic compounds (e.g., 4‐NP to 4‐AP) in water using NaBH_4_ (Table 5). It demonstrates high activity and selectivity. It also catalyzed the Suzuki reaction, showing significant efficiency in aqueous media. The catalysts were recovered after 10 cycles with minimal reduction in performance, thanks to the protective double‐shell architecture. Table 6 compares the catalytic performance of Au‐based catalysts in the hydrogenation of 4‐NP.

**TABLE 5:** Reduction of various nitroarenes by Fe_3_O_4_@RGO@Au@C CDSNs.

Entry	Substrate	Time, min	Yield, %
1	1‐Methoxy‐4‐nitrobenzene	150	74
2	Nitrobenzene	120	79
3	1‐Methyl‐4‐nitrobenzene	140	79
4	2‐NP	7	99
5	4‐NP	13	98
6	3‐NP	4	99

*Note:* Reaction condition: 1.20 × 10^−4^ M nitroaromatic compound (30 mL), 0.17 M NaBH_4_ (30 mL), and catalyst (2 mol% of the gold concentration).

**TABLE 6:** Comparison of catalytic performance by Au‐based catalysts in the hydrogenation of 4‐NP.

Entry	Catalyst	Catalysts, mol%	TOF, min^−1^	Ref.
1	GO‐Fe_3_O_4_‐Au NPs(G)	7.3	8.06	[67]
2	rGO/Fe_3_O_4_/Au	—	11.93	[68]
3	Au/graphene hydrogel	43	0.19	[69]
4	Au NPs/SNTs	28	0.77	[70]
5	Au@SiO_2_	7	0.45	[71]
6	Fe_3_O_4_@P(EGDMA‐co‐MAA)/Au	2.5	1.6	[72]
7	Fe_3_O_4_@SiO_2_‐Au@mSiO_2_	9	0.73	[73]
8	Au‐PMMA	7	1.23	[74]
9	HAuCl_4_·3H_2_O	46	0.11	[75]
10	Fe_3_O_4_@RGO@Au@C	2	3.77	Currently discussed report

Dr et al. described the synthesis, characterization, and catalytic application of a novel nanohybrid material consisting of ultrafine Pd NPs and magnetic Fe_3_O_4_ particles assembled on polyethyleneimine‐functionalized reduced graphene oxide (PEI/RGO) sheets [76]. The nanohybrid, denoted as Pd NPs/Fe_3_O_4_/PEI/RGO, was prepared by first forming PEI/RGO sheets, then loading with Pd NPs (average size ∼2.2 nm) and assembling Fe_3_O_4_ NPs (∼6 nm). PEI acted as the coupling agent, and formaldehyde (HCHO) is used as the reducing agent. TEM showed well‐dispersed, ultrafine Pd and Fe_3_O_4_ NPs on RGO sheets. XPS and XRD verified composition and crystallinity. EDX, ICP‐AES determined elemental content (Pd loading ∼1.06%; Fe ∼2.02%). The hybrid catalyst (Pd NPs/Fe_3_O_4_/PEI/RGO) exhibited high activity for converting 4‐NP to 4‐AP in H_2_O, using NaBH_4_ as the reducing agent. Over 92% conversion was achieved in 6 min at room temperature. The reaction followed first‐order kinetics with a rate constant of 0.1691 min^−1^. Catalytic performance improved with increased NaBH_4_ or catalyst dosage. The RGO supports increased dispersion and stability of Pd NPs and offers high adsorption of 4‐NP *via* *π*‐*π* interactions, enhancing local concentration and reaction rates. The Fe_3_O_4_ phase allowed for magnetic separation of the catalyst post‐reaction. The system harnesses the synergistic effects of Pd NPs and RGO for electron transfer, thereby boosting reduction efficiency. This nanocatalyst was reused for 10 cycles with no substantial drop in catalytic performance or structural integrity, as confirmed by TEM after cycling. Magnetic recovery was rapid and convenient, minimizing catalyst loss. This system not only exhibited superior catalytic activity for environmental remediation (4‐NP reduction) but also solved common issues of nanoparticle aggregation and separation. It has potential applications across catalysis, the environment, biotechnology, and energy.

**SCHEME 4:**
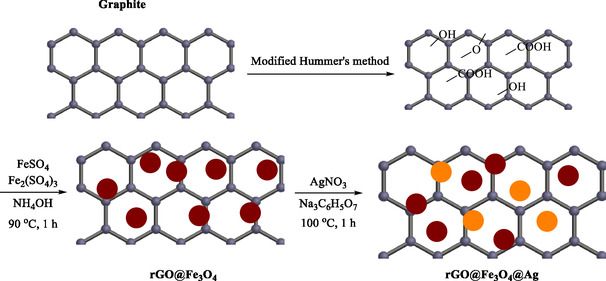
Synthesis of rGO/Fe_3_O_4_/Ag NH.

In 2017, Thu et al. explained the eco‐friendly preparation of a reduced graphene oxide/Fe_3_O_4_/Ag (rGO/Fe_3_O_4_/Ag). They demonstrated its effectiveness as a retrievable nanocatalyst for the reduction of 4‐NP, a common environmental pollutant [77]. The rGO/Fe_3_O_4_/Ag nanohybrid was produced *via* a simple, eco‐friendly method utilizing sodium citrate as the reducing and capping agent. GO was first synthesized using a modified Hummers’ method, Fe_3_O_4_ NPs were deposited on GO via co‐precipitation, and Ag NPs were then reduced and deposited onto the rGO/Fe_3_O_4_ composite (Scheme 4). The resulting rGO/Fe_3_O_4_/Ag nanohybrid was analyzed using TEM, SEM‐EDX, XRD, FT‐IR, Raman, and VSM. This nanohybrid contains ∼13.5% rGO, 62.5% Fe_3_O_4_, and 24% Ag by mass. Fe_3_O_4_ NPS (8–16 nm) and Ag NPS (18–40 nm) are densely distributed on rGO sheets. The composite exhibited superparamagnetic properties (saturated magnetization 29 emu/g), facilitating easy magnetic separation and reuse.

The rGO/Fe_3_O_4_/Ag nanohybrid served as a useful nanocatalyst for the hydrogenation of 4‐NP to 4‐AP using NaBH_4_. This reaction rate constant is 0.37 min^−1^, and the half‐life for 4‐NP reduction was about 1.86 min, comparable to those of other Ag‐based nanocatalysts (Table 7). The catalyst retained 94.4% conversion efficiency after four cycles due to its magnetic recoverability.

**TABLE 7:** Comparison of rate constants for different Ag‐based nanocatalysts.

Entry	Catalyst	**Apparent rate constant, min** ^**−1**^	Ref.
1	Ag dendrites	0.34	[78]
2	rGO/Fe_3_O_4_/Ag	0.25–0.69	[79]
3	rGO/Ag	0.49	[80]
4	rGO/Fe_3_O_4_/Ag	0.85–1.60	[81]
5	Fe_3_O_4_/Ag	0.24	[82]
6	PSMAA/Ag	0.19–0.49	[83]
7	PG/Ag	0.33	[84]
8	rGO/Fe_3_O_4_/Ag	0.37	Currently discussed report

**SCHEME 5:**
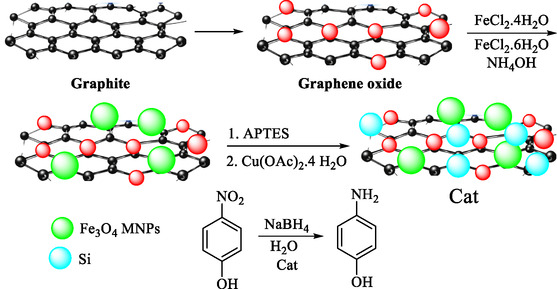
Preparation process and application of Fe_3_O_4_/GO/Pr‐NH_2_‐Cu(II) in the reduction of 4‐NP.

Zabihzadeh et al. reported the preparation, characterization, and uses of a new magnetically recyclable nanocomposite (Fe_3_O_4_/GO/Pr‐NH_2_‐Cu(II)) as an effective, reusable nanocatalyst in the hydrogenation of toxic 4‐NP to 4‐AP in water [85]. GO was synthesized using a modified Hummers method. Fe_3_O_4_ NPs were anchored on GO to prevent aggregation and provide anchoring sites. The composite was further modified with 3‐aminopropyltriethoxysilane (APTES) and finally coordinated with Cu(OAc)_2_, yielding Fe_3_O_4_/GO/Pr‐NH_2_‐Cu(II) (Scheme 5).

Some analytical techniques, including FT‐IR, XRD, FE‐SEM, EDX, ICP‐OES, VSM, and UV‐Vis, were used to characterize this nanocatalyst. The FE‐SEM image showed granular nanoparticles with diameters of 18–28 nm. EDX analysis confirms the existence of O, Fe, C, Si, N, and Cu, indicating successful loading. ICP‐OES reveals 6.3% copper content. VSM Magnetization (26.9 emu/g) was sufficient for easy magnetic separation. XRD data confirms the crystalline structure of Fe_3_O_4_ and loss of stacking, indicating good nanocomposite formation. FT‐IR showed characteristic bands of GO, Fe_3_O_4_, APTES, and Cu(II) complex. The nanocomposite efficiently catalyzed the hydrogenation of 4‐NP to 4‐AP in H_2_O at 25°C within 4 min using NaBH_4_ as a reducing agent. UV‐vis spectroscopy tracked conversion; complete reaction observed within 4 min at room temperature, which was rapid compared to many reported catalysts (Table 8). The catalyst promoted electron transfer from BH_4_^‐^ to 4‐NP, enabling reduction through *π*–*π* stacking and surface adsorption. This catalyst was recoverable and reusable for five runs without significant loss of activity. Advantages of this method include fast reaction time, use of green, inexpensive materials, stability, and ease of recovery.

**TABLE 8:** Comparison of different catalysts in the hydrogenation of 4‐NP.

Entry	Catalyst	Catalyst loading	Time, min	Ref.
1	Ni‐PVAm/SBA‐15	12 mg	85	[86]
2	Au@TpPa‐1	20 mg	13	[75]
3	Hypercross‐linked porous PS/IL networks	150 mg	10	[87]
4	Pd@HCS	3 mg	5	[88]
5	Ag/KCC‐1	20 µ L, 10 mg	8.5	[89]
6	Cu microspheres	5 mg	18	[90]
7	Au‐Ag bimetallic NPs supported on LDH	1 µ L, 1 mg	30	[20]
8	Au/graphene hydrogel	0.1 mg	12	[69]
9	Fe_3_O_4_/GO/Pr‐NH_2_‐Cu(II)	10 mg	4	Currently discussed report

Parrott and Erasmus reported the synthesis, characterization, and catalytic performance of four Pd‐containing GO nanocomposites, with combinations of CNT and Fe_3_O_4_ (Pd/CNT/Fe_3_O_4_/GO, Pd/CNT/GO, Pd/Fe_3_O_4_/GO, and Pd/GO) [91]. GO was prepared by oxidizing graphite (modified Hummer's method). Fe_3_O_4_ NPs and CNTs were incorporated into GO, followed by in situ deposition of Pd NPs. Various techniques were used for characterization of the nanocatalyst, including FTIR, PXRD, TGA, TEM, SEM, ICP‐OES, and XPS. FTIR and PXRD analysis confirmed the successful oxidation of graphite to GO, with increased interlayer spacing and the formation of oxygen functional groups. SEM and TEM images demonstrated well‐dispersed, mostly globular nanoparticles with diameters ≤32 nm. Pd NPs averaged 11–17 nm, depending on the composite. TGA data showed improved thermal stability in nanocomposites compared to pure GO. ICP‐OES verified Pd content ranged from 7.9% to 9.1% by mass. XPS data determined elemental compositions and Pd oxidation states (Pd^0^:Pd^2+^ ratios). Hydrogenation of three NP isomers (4‐NP, 3‐NP, and 2‐NP) to their corresponding AP was performed, utilizing NaBH_4_ as reductant. The highest TOF for the hydrogenation of 4‐NP and 2‐NP was achieved with Pd/CNT/Fe_3_O_4_/GO, which outperformed other catalysts. For 3‐NP, Pd/GO gave the highest TOF. Adding Fe_3_O_4_ to GO reduced activity unless combined with CNT. CNT increased catalytic activity and presumably improved the accessibility and electron transfer to Pd NPs. Catalytic efficiency correlated positively with the proportion of metallic Pd^0^; Pd^0^ was significantly more active than Pd^2+^. Reduction of Pd^2+^ to Pd^0^ was observed during initial catalytic cycles. Some catalysts (especially Pd/GO and Pd/Fe_3_O_4_/GO) showed increased activity in the second cycle due to increased Pd^0^ content. Incorporating Pd, CNT, and Fe_3_O_4_ improved composite stability, with mass losses under 5% up to 120°C; pure GO decomposed more. The strategic incorporation of CNT and Fe_3_O_4_ into GO‐based nanocomposites allowed for both magnetic recovery (due to Fe_3_O_4_) and catalytic enhancement (due to CNT spacing and electron transfer). Optimizing the Pd^0^/Pd^2+^ ratio was crucial, as Pd^0^ was the active species for nitrophenol reductions. The Pd/CNT/Fe_3_O_4_/GO composite is the most effective overall. There was a direct proportional relationship between the Pk_a_ of the nitrophenol substrate and the catalyst's initial TOF for certain composites. These nanocomposite catalysts showed promise for the environmental remediation of nitrophenolic pollutants, offering both high activity and recyclability.

In 2021, Tung et al. reported the development and characterization of Pd‐containing nanocomposites, particularly those containing chitosan (Chit), GO, CNT, and Fe_3_O_4_. These nanocomposites were designed to serve as effective, recyclable nanocatalysts for the hydrogenation of nitrophenolic compounds (pollutants) in water [92]. Chit was crosslinked with GO using glutaraldehyde to enhance its mechanical stability and create a basic nanocomposite support. Further modifications included incorporating CNTs (to improve GO sheet separation and electron transfer) and magnetite NPs (to impart magnetic properties for easy catalyst recovery). Pd NPs were embedded in these nanocomposites to act as the active catalytic component. Advanced techniques, including ATR‐FTIR, TEM, SEM, TGA, XPS, and ICP‐OES, were used to characterize the structure, morphology, elemental composition, thermal properties, and oxidation states. Characterization confirmed successful incorporation of all components and uniform dispersion of Pd NPs. The nanocomposites were evaluated for their catalytic activity in reducing three different nitrophenols (2‐, 3‐, and 4‐ NP) in H_2_O, using NaBH_4_ as the reducing agent. All nanocomposites showed high efficiency, following pseudo‐first‐order kinetics. The presence of CNT improved the hydrophilic/hydrophobic balance and catalysis in water.

The incorporation of Fe_3_O_4_ enabled magnetic separation and catalyst reuse. The rate constants (k´obs) for reduction were in the range of 2.0 × 10^−3^ to 2.5 × 10^−2^ s^−1^, higher than several related catalysts reported in the literature. Table 9 shows a comparison of the rate constant k’obs (s^−1^) for the hydrogenation of 4‐NP over Pd nanocatalysts on various supports. Notably, the Pd/Chit/GO/CNT/Fe_3_O_4_ nanocomposite exhibited superior turnover numbers (TON) and turnover frequencies (TOF), indicating greater catalytic efficiency with lower Pd content. The catalyst could be recycled multiple runs with negligible reduction of performance (86%–98% effectiveness in the second cycle). Chit served as both a stabilizer and an electronic donor, improving the stability and catalytic efficiency of Pd nanoparticles. The synergy among GO, CNT, Fe_3_O_4_, and Pd NPs created an effective platform for electron transfer and pollutant reduction. The study demonstrates that GO‐based nanocomposites, further modified with CNT and magnetite, are promising, green, and economical catalysts for removing nitrophenolic pollutants from water. Magnetic properties allow for easy recovery and reuse. The platform offers opportunities to design versatile, sustainable catalysts for a range of environmental and chemical processes.

**TABLE 9:** Comparison of the rate constant k’obs (s^−1^) for the hydrogenation of 4‐NP over Pd nanocatalysts on various supports.

Samples	**k’obs, s** ^**−1**^	Ref.
Pd nanocrystals	4.83 × 10^−3^	[93]
Pd/FG	2.35 × 10^−3^	[94]
Pd NPs@Fe_3_O_4_/Cs‐AG microcapsules	5.1 × 10^−3^	[95]
Pd/microgel‐PS	1.50 × 10^−3^	[96]
CNT/PiHP/Pd	1.67–5.0 × 10^−3^	[97]
Pd/Al_2_O_3_	9.2 × 10^−3^	[98]
Pd NPs	0.02 × 10^−3^	[97]
Pd/Fe_3_O_4_@SiO_2_@KCC‐1	19.6 × 10^−3^	[99]
Pd/SBA‐15	11.8 × 10^−3^	[97]
Pf/MPC	12 × 10^−3^	[100]
**Pd/GO**	**11.3 × 10** ^**−3**^	**Currently discussed report**
**Pd/Chit/GO**	**25.2 × 10** ^**−3**^	**Currently discussed report**
**Pd/Fe**_**3**_ **O**_**4**_ **/Chit/GO**	**6.9 × 10** ^**−3**^	**Currently discussed report**
**Pd/CNT/Chit/GO**	**10 × 10** ^**−3**^	**Currently discussed report**
**Pd/CNT/Fe**_**3**_ **O**_**4**_ **/Chit/GO**	**6.9 × 10** ^**−3**^	**Currently discussed report**

**SCHEME 6:**
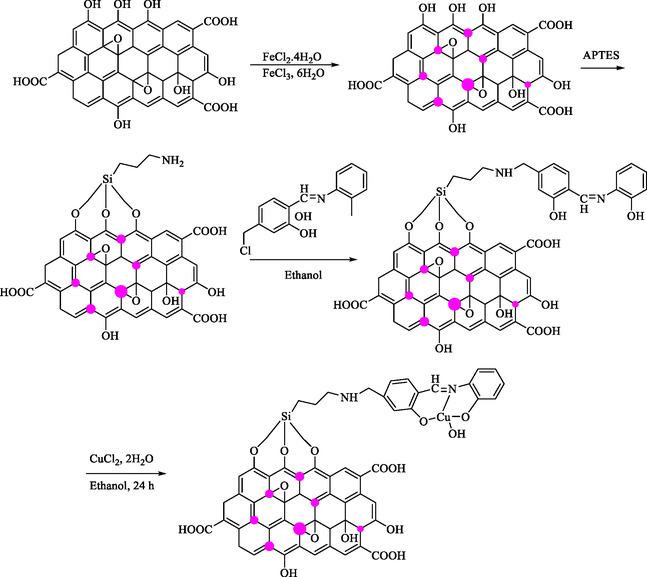
Schematic model for the preparation of GO/Fe_3_O_4_/CuL.

Dastjerdy and Monadi presented the design, synthesis, and application of a copper(II) Schiff base complex immobilized on magnetic GO (MGO) as a heterogeneous nanocatalyst. The main focus was on environmental remediation and the synthesis of valuable organic compounds [101]. The Cu(II) Schiff base complex was anchored to MGO via a stepwise chemical process. This involved functionalizing GO with amino‐silane groups, attaching the Schiff base ligand, and complexing it with Cu(II) ions (Scheme 6).

Comprehensive characterization was performed using FT‐IR, XRD, SEM, and TEM, confirming successful binding and morphology. TGA demonstrated the thermal stability of this nanocomposite. VSM verified the magnetic separability of the nanocatalyst, enabling easy recovery. ICP‐OES/EDS determined the copper content and elemental distribution. The Cu(II)‐Schiff base@MGO catalyst efficiently catalyzed the degradation of organic pollutants, especially nitrophenols and dyes, from water streams. It achieved high conversion rates and was effective across multiple cycles thanks to its magnetic recovery. The reaction efficiently catalyzed the hydrogenation of 4‐NP to 4‐AP utilizing NaBH_4_. High yields ( >90%) were achieved in short times (∼5–10 min). Catalyst could be reused for up to 8 cycles with negligible loss in activity. This research demonstrated that a Cu(II) Schiff base complex immobilized on MGO serves as a versatile, effective, and recyclable catalyst in environmental cleanup and organic synthesis. Its facile magnetic separation, high catalytic activity, and sustainability make it promising for practical applications in green chemistry and water treatment.

**SCHEME 7:**
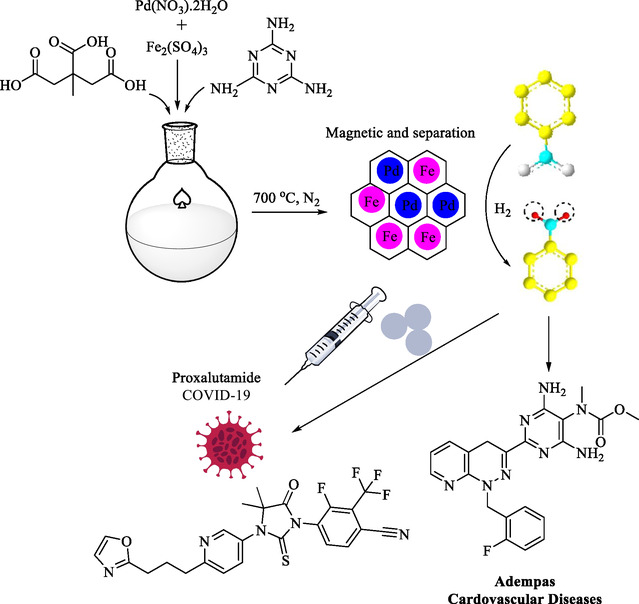
Preparation of Pd/Fe@N/C catalyst and its application in the synthesis of drugs Intermediate.

Lin et al. reported the development of a new bimetallic nanocatalyst, low‐loading Pd and Fe NPs encapsulated in nitrogen‐doped graphene on a magnetic carbon support (Pd/Fe@N/C) (Scheme 7), for the efficient reductive amination of nitroarenes under moderate conditions [102]. The Pd/Fe bimetallic nanoparticles were prepared and encapsulated within a nitrogen‐doped graphene carbon shell. The structure provided a high density of active sites and robust stability. Nitrogen doping created additional anchoring and electron‐rich sites, thereby enhancing metal dispersion and catalytic activity. The encapsulating graphene layer protects the nanoparticles and improves stability, while Fe provides magnetism and may enhance synergistic catalysis.

XRD confirmed the formation of thin graphene shells and the presence of Pd, Fe_3_O_4_, and *N*‐doped carbon. Magnetic properties were confirmed, allowing for easy solid‐phase recovery and reuse of the catalyst. This catalyst contains a low Pd content of 2.35%. This Pd/Fe@N/C nanocatalyst demonstrated outstanding catalytic performance, achieving a nitrobenzene conversion of 99% and aniline selectivity of 99% under mild conditions of 0.8 MPa hydrogen pressure and 40°C. The process was highly selective, forming only the amine product with water as a byproduct; minimal or no side reactions were observed. Various aliphatic nitro and halogenated derivatives were well tolerated and successfully converted into the corresponding amines with high selectivity. Furthermore, this bimetallic nanocatalyst exhibited magnetic properties, making it easy to isolate, recover, and reuse without metal leaching. It also efficiently catalyzed the extraordinarily selective reductive amination of aromatics in a green and economical manner, with water as the sole byproduct.

**SCHEME 8:**
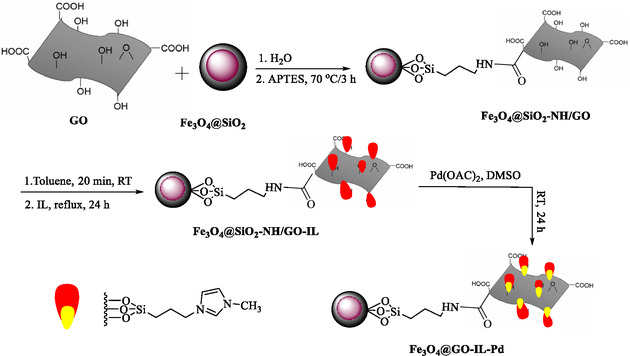
Preparation of Fe_3_O_4_/GO–IL–Pd.

In 2024, Dadvar and Elhamifar reported the development, preparation, characterization, and catalytic activity of a new nanocomposite, Fe_3_O_4_/GO modified with an ionic liquid and Pd (Fe_3_O_4_/GO‐IL‐Pd). The catalyst was developed as a green, effective, and magnetically retrievable system for the hydrogenation of nitrobenzenes to anilines [103]. GO was combined with amine‐functionalized Fe_3_O_4_@SiO_2_ nanoparticles. The composite was further functionalized by a trimethoxysilylpropyl imidazolium chloride ionic liquid (IL). Pd was immobilized onto the IL‐modified Fe_3_O_4_/GO (Scheme 8). This resulted in a magnetically separable, high‐surface‐area nanocomposite with Pd catalytic sites stabilized by the IL. Comprehensive analyses confirm the structure and properties of this Fe_3_O_4_/GO‐IL‐Pd nanocatalyst.

FTIR and PXRD confirmed the presence of the expected functional groups and crystal phases. SEM, EDS, and EDS‐mapping reveal morphology (layered GO with uniform Fe_3_O_4_@SiO_2_ spheres) and uniform distribution of Pd. VSM confirmed strong magnetic properties, allowing for easy catalyst separation. TGA demonstrated high thermal stability. ICP‐OES showed Pd loading is 0.29 mmol/g. Optimized conditions for the reduction of nitrobenzenes to anilines included 50 mg of catalyst (1.45 mol% Pd), water as the solvent, room temperature, and NaBH4 as the reducing agent (Table 10). It reduces various substituted nitrobenzenes (electron‐donating and ‐withdrawing groups) to anilines in high yield (89%–96%) within 10–15 min. Catalyst was easily recovered with a magnet and reused seven times with negligible reduction in performance and stability. The Leaching Test showed no Pd leaching, demonstrating the high stability of the immobilized Pd. The PXRD, SEM, and EDS confirmed structural and chemical integrity after multiple cycles. The proposed mechanism involved adsorption of BH_4_^‐^ ions onto the nanocomposite surface, with Pd mediating electron transfer from NaBH_4_ to nitroarene, leading to reduction. The IL stabilizes Pd and prevents agglomeration or leaching during catalysis. This research presented a robust, green, magnetically separable Pd nanocatalyst based on ionic‐liquid‐modified Fe_3_O_4_/GO for rapid and efficient reduction of nitrobenzenes to anilines. The system suggests high yields, sustainability, and operational simplicity, and makes a significant contribution to green catalytic methodologies for aromatic amine synthesis. The catalytic activity of Fe_3_O_4_/GO‐IL‐Pd and previously reported catalysts for the hydrogenation of nitrobenzene is shown in Table 11.

**TABLE 10:** Reduction of nitrobenzene compound using the Fe_3_O_4_/GO‐IL‐Pd nanocomposite.

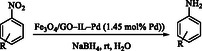					
Entry	R	Time, min	Yield, %	**TON, min** ^**−1**^	**TOF, min** ^**−1**^
1	H	10	95	65.51	409.43
2	4‐NH_2_	15	95	65.51	262.04
3	2‐OH	10	89	61.37	383.56
4	4‐OH	10	96	66.20	413.75
5	3‐CHO	15	95	65.51	262.04

*Note:* Reaction conditions: nitrobenzene (1 mmol), NaBH_4_ (2 mmol), nanocatalyst (1.45 mol% Pd), and water (5 mL), 25°C.

**TABLE 11:** Comparison of the catalytic activity of Fe_3_O_4_/GO‐IL‐Pd and formerly reported catalysts for the hydrogenation of nitrobenzene.

Catalyst	Conditions	Yield, %	Recovery cycles	Ref.
GA‐Pd/ZnO	H_2_ atmosphere, RT, 2 h, catalyst (10 mg)	98	4	[104]
NiNPs/DNA	H_2_O, NaBH_4_, RT, 2 h, catalyst (2 mol%)	99	4	[105]
Fe_3_O_4_@Cur/Mel‐Ag	NaBH_4_ (2.0 mmol)/40 mg K_2_CO_3_/pH = 8, 70°C, 10 min, catalyst (20 mg)	98	4	[106]
CuO‐GO	NaBH_4_, RT, 30 min, catalyst (50 mg)	98	5	[107]
Fe_3_O_4_/GO‐IL‐Pd	**H**_**2**_ **O, NaBH**_**4**_ **, RT, 10 min, catalyst (50 mg)**	**95**	**7**	**Currently discussed report**

**SCHEME 9:**
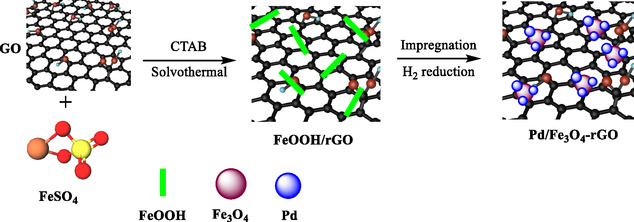
Schematic illustration for the preparation procedure of Pd/Fe_3_O_4_‐rGO.

In 2025, Wang et al*.* presented the development, preparation, and catalytic uses of a new Pd/Fe_3_O_4_‐rGO nanocomposite for the highly chemoselective hydrogenation of nitroaromatic compounds [108]. The catalyst was prepared by depositing Pd NPs onto a composite support comprising Fe_3_O_4_ and rGO (Scheme 9). Special attention was given to optimizing the interface between Pd, Fe_3_O_4_, and rGO to maximize Pd NPs dispersion and interaction with the dual support. The inclusion of Fe_3_O_4_ imparted magnetic properties, enabling easy separation and recyclability of the catalyst after reaction.

The catalyst was characterized using advanced techniques, including TEM, XRD, SEM, and XPS. Highly dispersed, stable Pd nanoparticles were achieved due to the engineered interface between the components. The catalyst efficiently promoted the hydrogenation of nitroaromatic compounds to their corresponding anilines under mild conditions. Exceptional chemoselectivity was observed, meaning functional groups (e.g., halogens, carbonyls) on the nitroaromatic substrate are retained without reduction or dehalogenation. High conversion rates and turnover frequencies were reported; the catalyst was effective even at low Pd loadings. The Pd/Fe_3_O_4_‐rGO nanocatalyst was magnetically recovered after multiple catalytic runs, with negligible loss in performance and selectivity. The interfacially engineered Pd/Fe_3_O_4_‐rGO nanocatalyst presents outstanding selectivity, activity, and recyclability for nitroaromatic hydrogenation. Its practical advantage stemmed from its high efficiency and magnetic recoverability, making it viable for scalable, sustainable environmental or industrial applications.

## 3 Conclusions

Recent advancements in Fe_3_O_4_@GO nanocatalysts have demonstrated their significant potential as effective, sustainable, and reusable systems in the hydrogenation of nitroarenes. These hybrid nanocatalysts combine the superparamagnetic behavior of Fe_3_O_4_ with the high surface area, thermal stability, and functional versatility of graphene oxide, resulting in enhanced catalytic performance under mild reaction conditions. Their ability to operate with green reducing agents such as NaBH_4_ or H_2_ makes them highly suitable for eco‐friendly chemical processes. Moreover, their easy magnetic recovery and reusability minimize catalyst loss and metal contamination, addressing key industrial and environmental concerns. The ongoing development of these nanocatalysts, focusing on improving selectivity, stability, and scalability, positions them as promising candidates for future applications in green chemistry, pharmaceuticals, and environmental remediation.

## 4 Outlook

The advancement of Fe_3_O_4_@GO magnetic nanocatalysts represents a significant step toward achieving greener, more efficient pathways for nitroarene reduction, a key transformation in pharmaceutical, agrochemical, and materials synthesis. As researchers continue to refine these systems, future efforts will likely focus on: (**a**) enhancing catalytic selectivity and TOF through surface engineering and functional group modifications of graphene oxide. (**b**) Improving long‐term stability and minimizing metal leaching to meet industrial reusability standards. (**c**) Scaling up synthesis methods while maintaining uniform dispersion of active sites to ensure reproducibility and performance on a larger scale. (**d**) Expanding the substrate scope to include more complex and sensitive nitroarenes could potentially broaden their application in fine chemical and drug production. (**e**) Integrating with renewable reducing agents or green energy sources (e.g., photothermal or electrochemical systems) to further align with sustainable chemistry principles. With their combined advantages of high efficiency, low toxicity, magnetic recoverability, and adaptability, Fe_3_O_4_@GO nanocatalysts are well‐positioned to play a central role in next‐generation catalytic technologies for eco‐friendly chemical transformations. Continued interdisciplinary research will be essential to fully unlock their potential in both academic and industrial settings. Fe_3_O_4_@GO nanocatalysts for nitroarene reduction, while exhibiting excellent catalytic efficiency and recyclability, face several current challenges that limit broader industrial application. Fe_3_O_4_@GO nanocatalysts face challenges during nitroarene hydrogenation, including nanoparticle aggregation, metal leaching, and reduced magnetization. These issues impact long‐term recyclability and activity retention. GO sheets tend to restack via *π*–*π* interactions and van der Waals forces, burying active sites and hindering substrate access. Fe_3_O_4_ nanoparticles aggregate without proper spacing, leading to uneven Pd dispersion and sintering under reaction conditions. Noble‐metal leaching (e.g., Pd) in aqueous media contaminates products and causes deactivation over cycles. GO functionalization helps, but strong reductants like NaBH_4_ accelerate this in prolonged use. Thick GO layers or multiple coatings lower saturation magnetization (often < 50 emu/g), complicating separation at scale. Balancing catalytic support with magnetic strength remains critical. Substrate selectivity decreases for sterically hindered nitroarenes, and scalability is limited by variability in hydrothermal synthesis. Process engineering strategies, such as continuous‐flow reactors paired with magnetic separation, enhance catalyst recovery, scalability, and sustainability. Advanced regeneration techniques, such as mild chemical treatments and ultrasonic dispersion, extend catalyst lifespan and maintain activity. These approaches enable greener, cost‐effective, and scalable nitroarene reduction using Fe_3_O_4_@GO nanocatalysts.

## Author Contributions

**Sara Payamifa**r**:** investigation, writing – original draft, visualization. **Majid Abdouss** **:** supervision. **Hamideh Sarreshtehdar Aslaheh** **:** writing – review and editing, software, conceptualization. **Ahmad Poursattar Marjani** **:** investigation, supervision, writing – review and edition.

## Conflicts of Interest

The authors declare no conflicts of interest.
